# Estimating pulsatile ocular blood volume from intraocular pressure, ocular pulse amplitude, and axial length

**DOI:** 10.1371/journal.pone.0283387

**Published:** 2023-03-23

**Authors:** Ryan H. Somogye, Cynthia J. Roberts, Eberhard Spoerl, Karin R. Pillunat, Lutz E. Pillunat, Robert H. Small

**Affiliations:** 1 Department of Biomedical Engineering, The Ohio State University, Columbus, OH, United States of America; 2 Department of Ophthalmology & Visual Sciences, The Ohio State University Wexner Medical Center, Columbus, OH, United States of America; 3 Department of Ophthalmology, Universitatsklinikum Carl Gustav Carus, Dresden, Germany; 4 Department of Anesthesiology, The Ohio State University Wexner Medical Center, Columbus, OH, United States of America; Bascom Palmer Eye Institute, UNITED STATES

## Abstract

The purpose of this study was to develop a method of estimating pulsatile ocular blood volume (POBV) from measurements taken during an ophthalmic exam, including axial length and using a tonometer capable of measuring intraocular pressure (IOP) and ocular pulse amplitude (OPA). Unpublished OPA data from a previous invasive study was used in the derivation, along with central corneal thickness (CCT) and axial length (AL), as well as IOP from the PASCAL dynamic contour tonometer (DCT) and intracameral (ICM) measurements of IOP for 60 cataract patients. Intracameral mean pressure was set to 15, 20, and 35 mmHg (randomized sequence) in the supine position, using a fluid-filled manometer. IOP and OPA measurements were acquired at each manometric setpoint (DCT and ICM simultaneously). In the current study, ocular rigidity (OR) was estimated using a published significant relationship of OR to the natural log of AL in which OR was invasively measured through fluid injection. Friedenwald’s original pressure volume relationship was then used to derive the estimated POBV, delivered to the choroid with each heartbeat as a function of OR, systolic IOP (IOP_sys_), diastolic IOP (IOP_dia_), and OPA, according to the derived equation POBV = log (IOP_sys_/IOP_dia_) / OR. Linear regression analyses were performed comparing OPA to OR and calculated POBV at each of the three manometric setpoints. POBV was also compared to OPA/IOP_dia_ with all data points combined. Significance threshold was p < 0.05. OR estimated from AL showed a significant positive relationship to OPA for both DCT (p < 0.011) and ICM (p < 0.006) at all three manometric pressure setpoints, with a greater slope for lower IOP. Calculated POBV also showed a significant positive relationship to OPA (p < 0.001) at all three setpoints with greater slope at lower IOP, and a significant negative relationship with IOP_dia_. In the combined analysis, POBV showed a significant positive relationship to OPA/ IOP_dia_ (p < 0.001) in both ICM and DCT measurements with R^2^ = 0.9685, and R^2^ = 0.9589, respectively. POBV provides a straight-forward, clinically applicable method to estimate ocular blood supply noninvasively. Higher IOP in combination with lower OPA results in the lowest values of POBV. The simplified ratio, OPA/ IOP_dia_, may also provide a useful clinical tool for evaluating changes in ocular blood supply in diseases with a vascular component, such as diabetic retinopathy and normal tension glaucoma. Future studies are warranted.

## Introduction

Ocular blood flow has been identified as a possible contributor to the pathogenesis of glaucoma, age-related macular degeneration (AMD), and ocular ischemic syndrome (OIS) [1]. In glaucoma, low ocular perfusion pressure (OPP, the driving force of ocular blood flow) has been identified as a risk factor [2]. Decreases or defects in choroidal blood flow have been linked by both theories and studies to the presence of AMD [1, 3]. Occlusion of the carotid or ophthalmic arteries result in reduced OPP, thus reduced occur blood flow, and are a leading cause of OIS [4, 5].

It has been shown that pulsatile ocular blood flow (POBF) can be estimated from intraocular pressure (IOP) and ocular pulse amplitude (OPA) if the ocular rigidity (OR), or pressure-volume relationship of the eye is known. This process requires analysis of a time-domain waveform of ocular pressure, usually produced by a pneumatonometer and post-processed numerically or graphically [6, 7]. Historically, the only studies to directly measure OR in an invasive procedure, have been on older subjects who are scheduled for ocular surgery to correct existing pathology, usually cataract surgery [8–11]. OR is measured directly by injecting a small, known amount of saline and measuring the pressure rise to give a subject-specific logarithmic pressure-volume relationship. This means the OR of younger, healthy eyes is not well characterized due to the inherent risks of measuring OR directly on otherwise healthy eyes and could bring into question the accuracy of POBF estimates for those subjects. This study presents a method for estimating subject-specific OR and calculating pulsatile ocular blood volume (POBV) with each cardiac cycle as an analysis tool for a variety of ocular diseases. OR has been shown to be highly correlated to axial length (AL) from an invasive study in cataract patients providing an equation to relate the two measurements [9]. OR estimated from the non-invasive AL measurement can be combined with IOP and OPA (replacing the time-domain waveform with the static components) to allow POBV to be estimated with each heartbeat.

## Methods

Unpublished OPA data originally acquired for a previous study on the accuracy of the PASCAL dynamic contour tonometer (DCT; Ziemer Ophthalmic Systems, Port, Switzerland) were used in the present study [12]. The original study was performed in accordance with the Declaration of Helsinki and was approved by the institutional ethics committee of the Medical Department of the University of Dresden. All patients signed written informed consent before entering the original study. Briefly, data from 60 patients were included in the current study (43 women, 17 men), all of whom underwent a complete ophthalmologic exam including central cornea thickness (CCT) measured by ultrasound pachymetry (Heidelberg Engineering, Heidelberg, Germany), corneal curvature measured by keratometry (Carl Zeiss Meditec, Inc., Dublin, CA), and AL measured by A-scan ultrasonography (Sonomed 2500; Technomed Maastricht, The Netherlands) before scheduled cataract surgery. The study hardware included a DCT tip integrated into the manometric pressure circuit for intracameral (ICM) measurements and a DCT attached to a Perkins handheld tonometer to facilitate transcorneal DCT measurements while the subject was supine. Once prepared for surgery but before the actual procedure, the subject corneas were cannulated and placed at three manometric IOP setpoints (15, 20, and 35mmHg). A stopcock was closed to isolate the tubing from the manometer prior to DCT and ICM measurements that were made simultaneously.

The volume of saline injected through the cannula to achieve the three IOP setpoints was not recorded so OR could not be calculated directly from the intracameral measurements, as in other studies [8, 10]. However, a previous invasive study (with similar subject demographics) directly calculating OR found a strong relationship (P < 0.001) of AL to the OR coefficient K [9]. Each subject’s OR was calculated using the first-order natural log fit line from Dastiridou et al., given in Eq 1 [9].

Eq 2 is Friedenwald’s original pressure volume relationship [13]. Solving for the volume change gives Eq 3 and inserting the corresponding pressures for a pulsatile IOP allows the POBV, units of μL entering the eye with each heartbeat to be calculated in Eq 4. It should be noted that traditionally IOP is the long-term average of ocular pressure with OPA being a pulsatile component that oscillates above and below IOP. However, DCT reports diastolic IOP so OPA is the pressure increase above the IOP reading [14]. Therefore, in the reported DCT values of the current study, DCT IOP = IOPdia in Eq 4. POBV does not have a time-dependent component and can be directly calculated from IOP, OPA, and OR coefficient K_AL_ (estimated from AL).


$$\begin{array}{l} K_{AL}=-0.0323\;\ln\left(AL\right)+0.1247\\ Where\;K_{AL}\;is\;the\;occular\;rigidity\;coeff,\;AL\;is\;axial\;length \end{array}$$ Eq 1

$$\begin{array}{l} Log(P/P_{0})=K_{AL}\times \Delta V\\ Where\;P_{0}\;is\;initial\;pressure,\;P\;is\;the\;inceased\;pressure,\;\Delta V\;is\;volume\;change \end{array}$$ Eq 2

$$\begin{array}{l} \Delta V=Log(P/P_{0})/\;K_{AL}=Log(\frac{IOPsys}{IOPdia})/\;K_{AL}\\ \mathrm{Where}\;{\mathrm{IOP}}_{\mathrm{sys}}=\mathrm{Systolic}\;\mathrm{IOP},\;\mathrm{and}\;{\mathrm{IOP}}_{\mathrm{dia}}=\mathrm{Diastolic}\;\mathrm{IOP};\;Solving\;for\;\Delta V \end{array}$$ Eq 3

POBV=ΔV=Log(OPA+IOPdiaIOPdia)KAL=Log(OPAIOPdia+1)(−0.0323ln(AL)+0.1247)Substituting(OPA+IOPdia)forIOPsys,andEq1forKALgivesPOBV Eq 4

## Results

**Fig 1:**
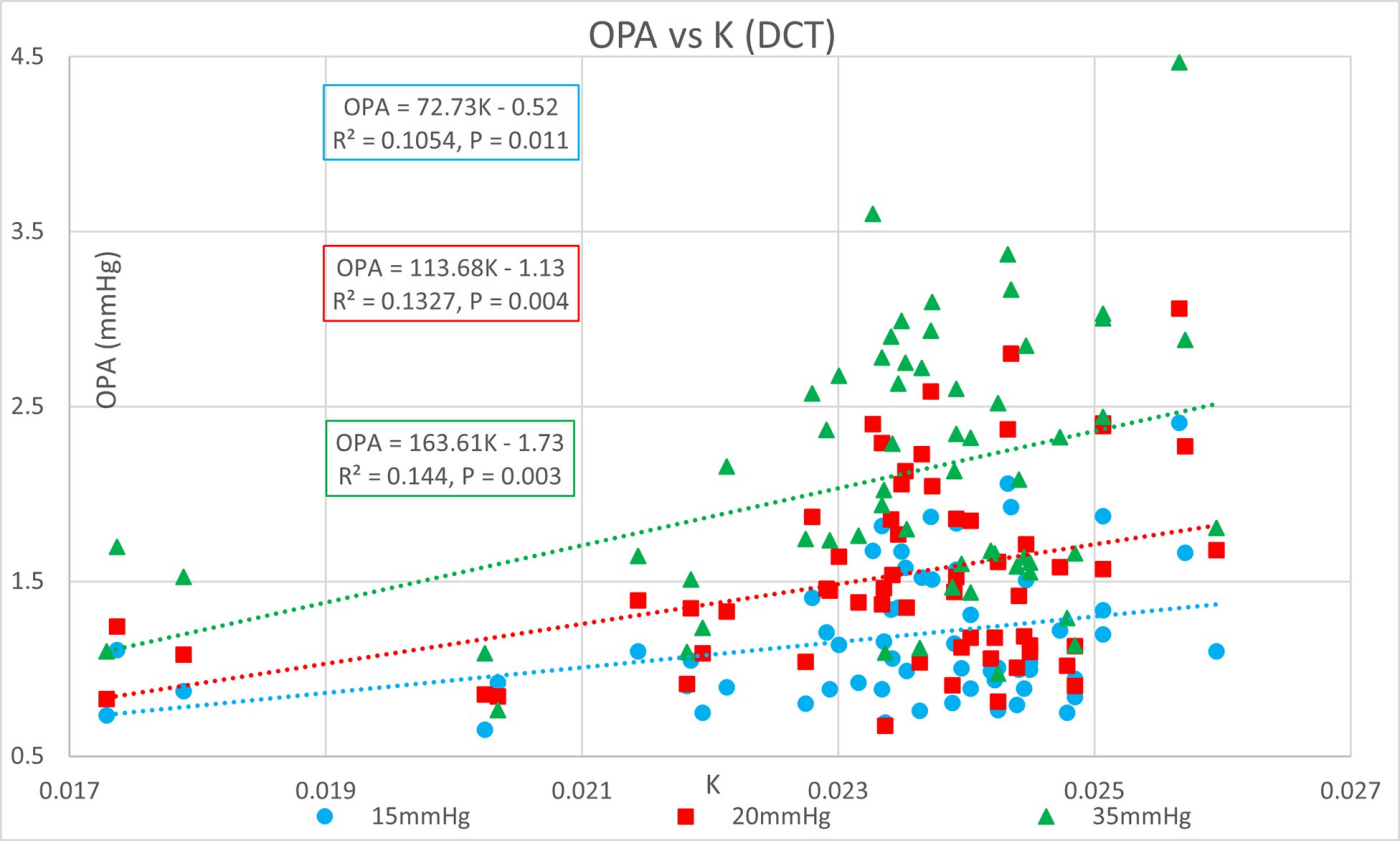
Positive correlation of OPA to estimated OR (K) for DCT measurements.

**Fig 2:**
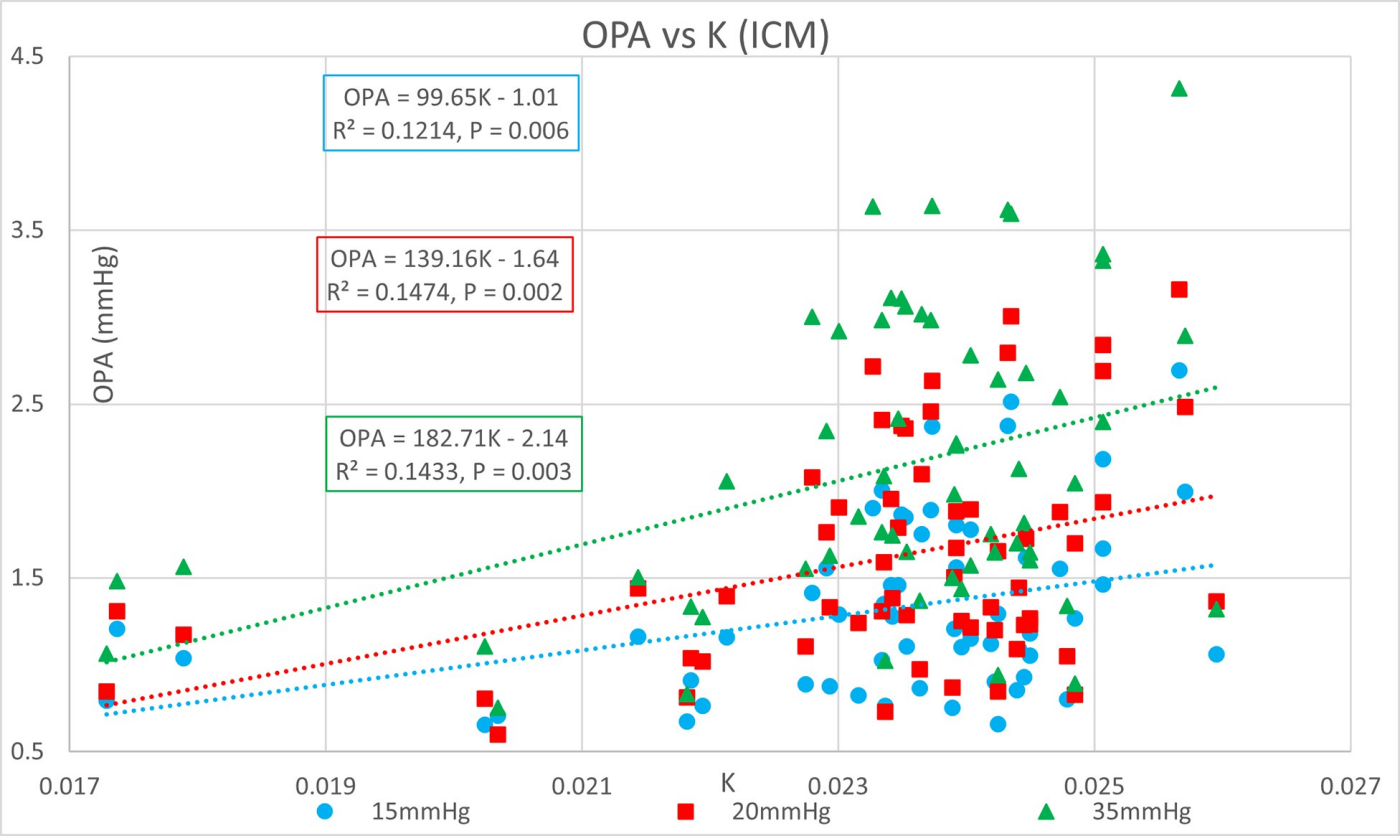
Positive correlation of OPA to estimated OR (K) for ICM measurements.

**Fig 3:**
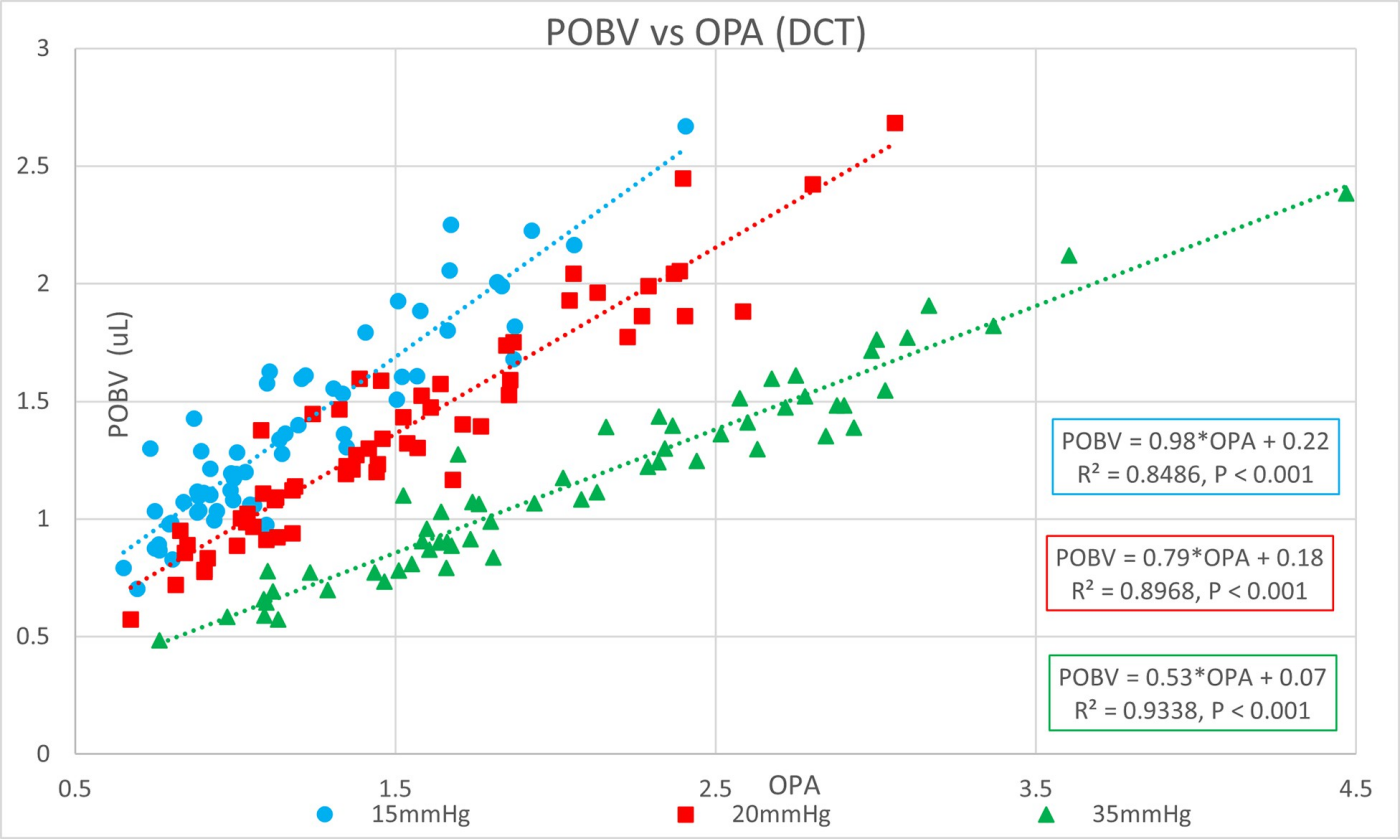
Positive correlation of POBV to OPA for DCT measurements.

**Fig 4:**
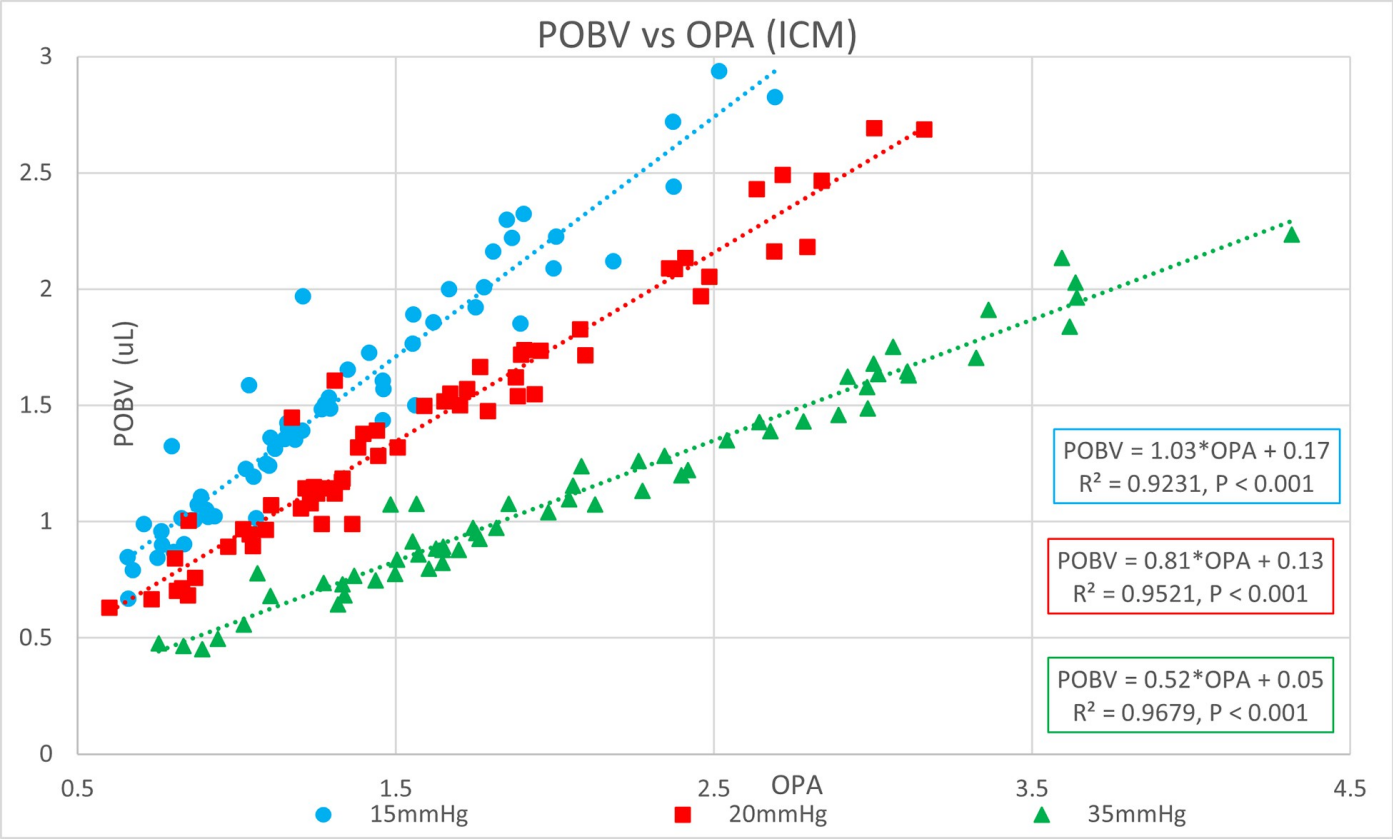
Positive correlation of POBV to OPA for ICM measurements.

**Fig 5:**
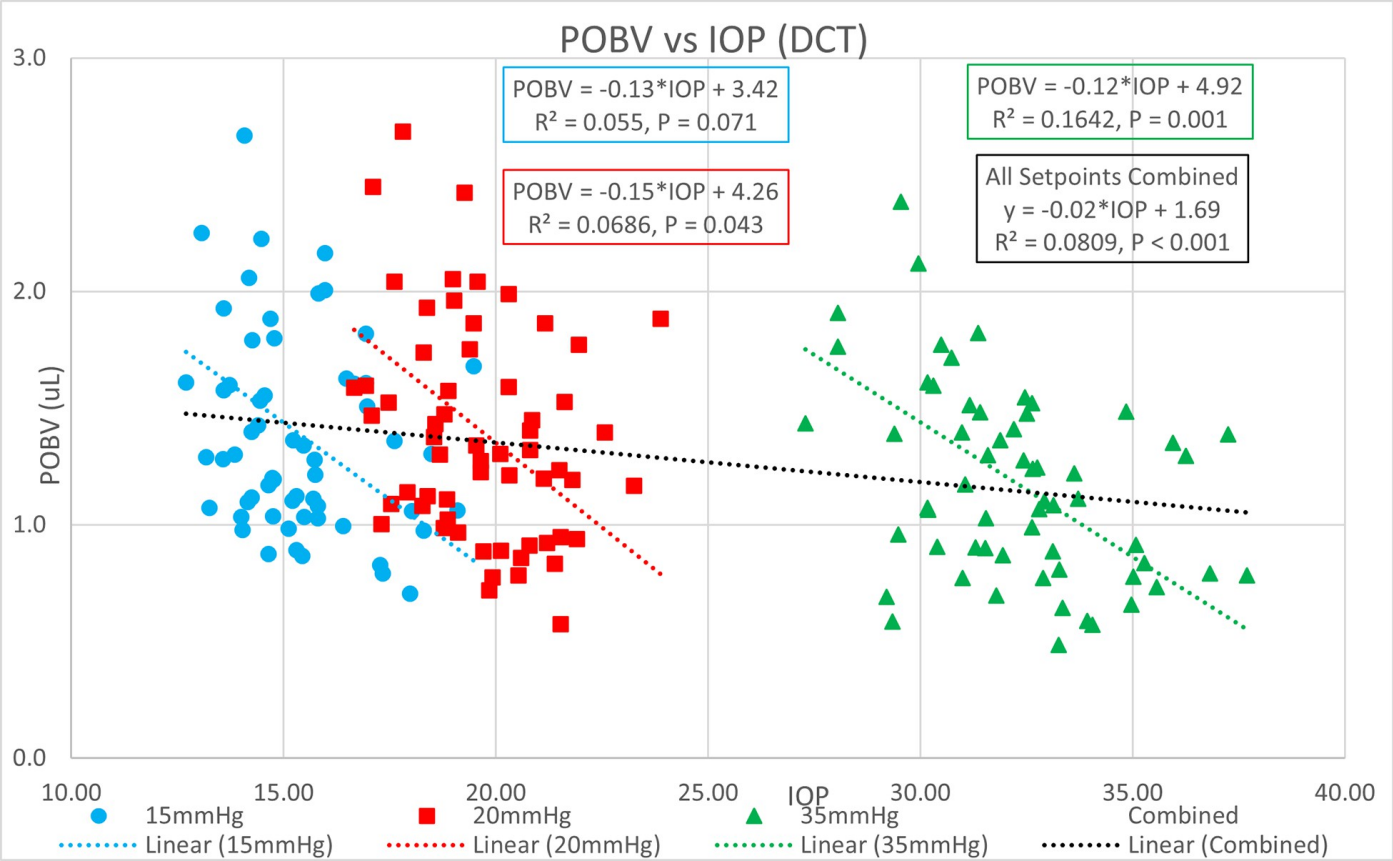
Negative correlation of POBV to IOP for DCT measurements.

**Fig 6:**
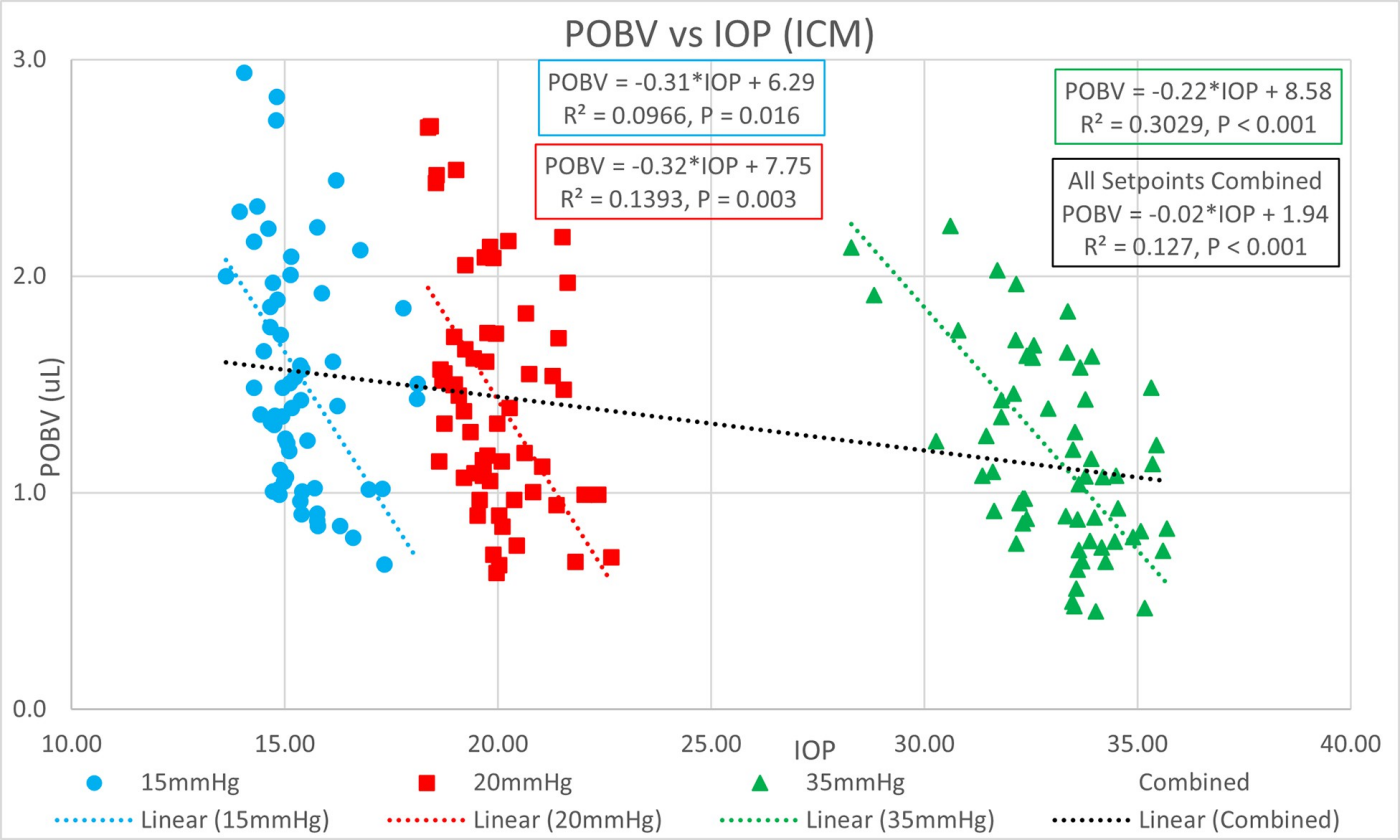
Negative correlation of POBV to IOP for ICM measurements.

**Fig 7:**
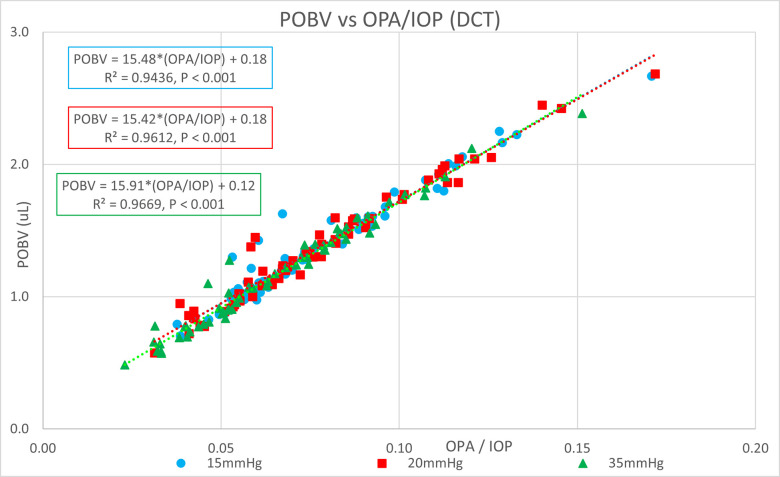
Strong correlation of the Calculated POBV to the Factor OPA/IOP for DCT Measurements.

**Fig 8:**
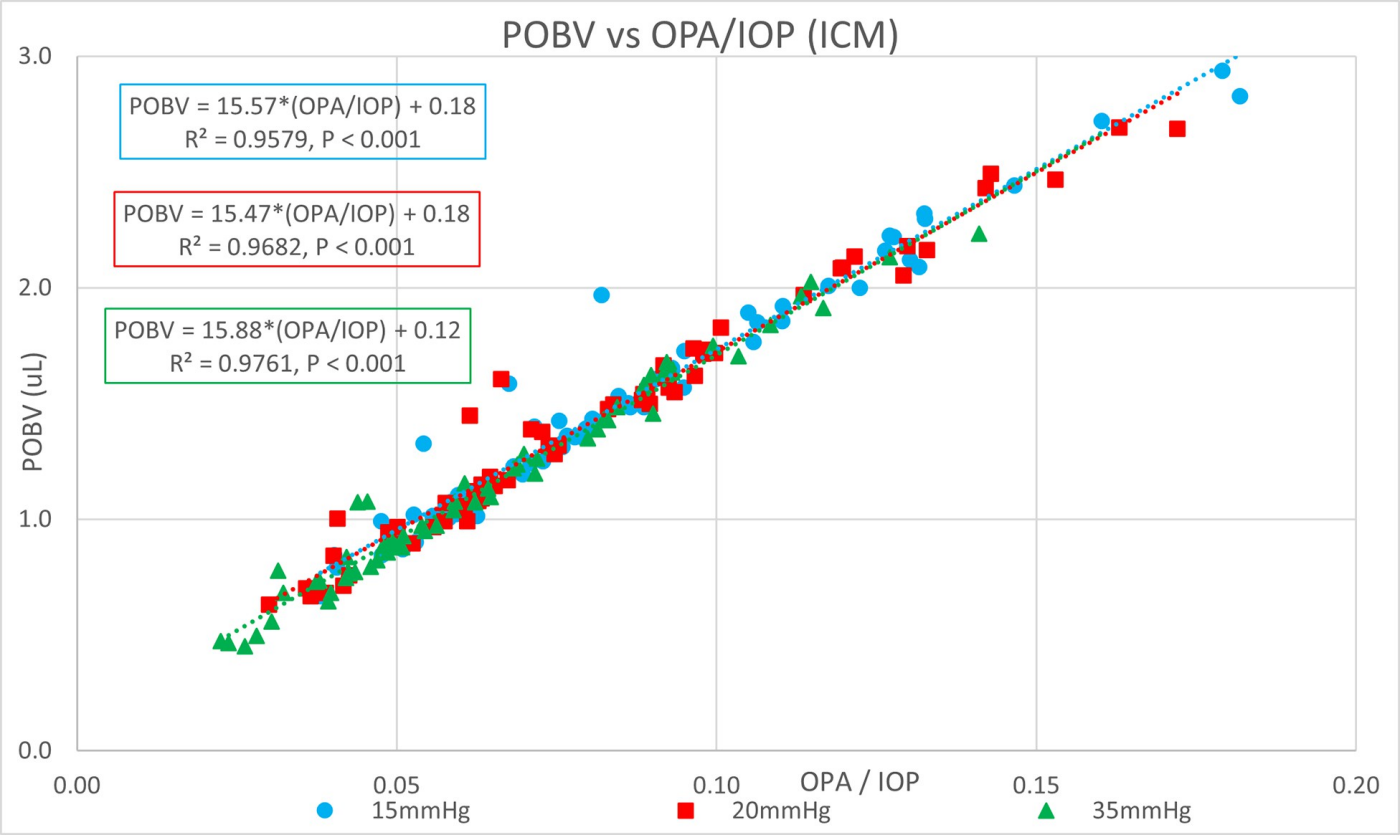
Strong correlation of the Calculated POBV to the Factor OPA/IOP for ICM Measurements.

Table 1 shows the ocular pressure measurement descriptive statistics of the original study. Table 2 shows the subject-specific ocular measurements made in the original study during a complete ophthalmic exam prior to the ocular pressure measurements and cataract surgery. The last column represents the estimated OR (K) calculated in the present study. Figs 1 and 2 show significant positive correlations of estimated OR (K) to OPA. Figs 3 and 4 show significant positive correlations of calculated POBV to OPA at all three setpoints, while Figs 5 and 6 show significant negative correlations of POBV and IOP at the two highest setpoints for DCT and all three for ICM. Figs 7 and 8 show significant correlations of POBV to the ratio of OPA / IOP, which is a surrogate factor of POBV if axial length is not available.

**Table 1:** Dataset IOP and OPA descriptive statistics.

	15mmHg Manometric				20mmHg Manometric				35mmHg Manometric			
All values mmHg	DCT	ICM	DCT	ICM	DCT	ICM	DCT	ICM	DCT	ICM	DCT	ICM
	IOP	IOP	OPA	OPA	IOP	IOP	OPA	OPA	IOP	IOP	OPA	OPA
**Min**	12.71	13.63	0.65	0.66	16.67	18.36	0.67	0.60	27.30	28.28	0.76	0.75
**Q1**	14.25	14.76	0.89	0.90	18.57	19.20	1.09	1.19	30.67	32.15	1.58	1.50
**Mean**	15.38	15.37	1.18	1.32	19.70	19.95	1.53	1.62	32.27	33.10	2.10	2.13
**Median**	15.16	15.11	1.05	1.19	19.60	19.78	1.43	1.42	32.32	33.50	1.98	1.92
**Q3**	16.08	15.76	1.43	1.63	20.82	20.49	1.86	1.94	33.42	34.00	2.69	2.90
**Max**	19.47	18.11	2.41	2.69	23.88	22.66	3.06	3.16	37.68	35.68	4.47	4.32
**SD**	1.56	0.98	0.40	0.51	1.63	1.04	0.55	0.64	2.31	1.54	0.76	0.85

**Table 2:** Subject measurements descriptive statistics.

	Age (yrs)	CCR K1 (Dpt)	CCR K2 (Dpt)	Mean K (Dpt)	Astig-matism (Dpt)	CCT (um)	Axial Length (mm)	Est. K(AL) (1/uL)
**Min**	49.00	39.90	40.30	40.58	0.00	464	21.27	0.0173
**Q1**	67.00	42.46	42.75	42.68	0.39	535	22.32	0.0231
**Mean**	73.40	43.45	43.89	43.67	1.03	562	23.05	0.0234
**Median**	75.00	43.60	43.85	43.65	0.80	563	22.79	0.0237
**Q3**	81.00	44.23	44.58	44.33	1.25	583	23.22	0.0244
**Max**	96.00	48.30	53.55	50.93	5.25	626	27.81	0.0259
**SD**	9.49	1.51	2.05	1.68	0.90	35.6	1.33	0.0018

## Discussion

Figs 1 and 2 show positive correlations of OPA to estimated OR for the present data set which is consistent with the literature using invasive methods to measure OR [8–10]. It should be noted that the slope of the regression line increased with the manometric IOP setpoint indicating the positive relationship of OPA to IOP. Figs 3 and 4 indicate positive correlations of POBV to OPA and a negative relationship of POBV to IOP since the slope of the regression line decreases with an increase in IOP setpoint. Figs 5 and 6 also show a significant negative correlation of POBV to IOP, for the two highest setpoints using DCT, but all three setpoints for ICM. This result is consistent with studies measuring POBF in normal subjects versus normal tension glaucoma subjects, which reported a negative correlation of POBF to IOP in both subject groups [15, 16]. Additional studies examining the validity of using time-varying measurements of IOP to calculate POBF show that OPA is directly related to POBF by way of the eye’s pressure-volume curve [6, 7]. This relationship supports findings that invasively increased IOP is positively correlated with OPA and negatively correlated to calculated POBF [8, 17]. Figs 7 and 8 show that POBV is also tightly associated with OPA/IOP, which is an excellent surrogate factor for POBF if axial length is not available. Although OPA is positively correlated with both POBV and IOP, IOP is negatively correlated with POBV, so dividing OPA by IOP has the effect of combining these opposite predictors of ocular blood volume. Therefore, an important contribution of the current work is that high IOP with low OPA is shown to be associated with the lowest values of POBV.

In the present study OR was estimated from AL based on an invasive study on cataract patients that found a strong negative correlation between OR and the natural log of AL [9]. This negative correlation between AL and OR is consistent with other studies, although not all showed significance [8, 10]. This may be due to the application of a linear regression fit line rather than the natural log of AL and/or the particular subject population under study. Ocular volume was found to be highly negatively correlated with OR in enucleated eyes [18]. While ocular volume can be estimated from AL, this doesn’t establish a direct correlation of AL to OR, since the pressure component is missing [19]. The positive correlation of OR to age has been established, meaning a limitation of this study is a lack of data on younger subjects which are often not included in invasive studies of OR since they have otherwise healthy eyes not scheduled for surgery [9, 20].

OIS occurs in patients with partial or total occlusion of the internal carotid artery on the same side as the affected eye. Poor collateral circulation between the internal and external carotid arteries usually accompanies the OIS diagnosis [4, 5]. OIS patients show a reduction of blood flow in the ophthalmic and retrobulbar arteries [21, 22]. In some cases ophthalmic artery flow direction is thought to be reversed, emptying into the lower-resistance intracranial arteries [22, 23]. Since the ophthalmic artery feeds the posterior ciliary arteries, which supply the choroid with blood, any reduction or reversal of ophthalmic artery flow would be expected to impact ocular blood flow substantially [24]. Quantifying ocular blood volume could provide insight into the level of carotid occlusion and overall cardiovascular health.

While the complete pathogenesis of glaucoma is unknown, reduced ocular blood flow has been associated with the progression of glaucoma, as well as age-related macular degeneration [1]. Glaucoma patients and hypertensive subjects have been shown to have significantly reduced POBF versus healthy controls [15, 17, 25–27]. Peripapillary retinal vessel diameter was significantly negatively correlated with the progression of glaucoma, independent of age in an age-matched prospective study of 473 eyes [28, 29]. Reduced peripapillary retinal blood flow and delayed choroidal filling was found in normal tension glaucoma (NTG) patients versus normal subjects, suggesting NTG patients experience reduced choroidal blood flow [30]. Delayed or altered choroidal filling measured by angiography, laser doppler flowmetry, and other techniques has been associated with AMD [31–33].

Sophisticated methods for measuring ocular blood flow have been developed, such as color Doppler imaging, Doppler Fourier domain optical coherence tomography, and laser speckle flowgraphy [34, 35]. However, the more fundamental method of interest is the calculation of POBF from time domain waveform of ocular pressure. This method relies on the eye pressure-volume relationship to translate the change in pressure into a change in volume. The derivative of the volume change represents POBF as a function of time [6]. Development of this method was based on the waveform output of a pneumatonometer. The DCT provides OPA and IOP but does not provide a time-domain waveform of ocular pressure. IOP, OPA, and an estimated OR can be used to estimate the volume of blood entering the eye noninvasively with each cardiac cycle, termed POBV.

Other limitations of this study relate to the data collection strategy of the original study. IOP was set manometrically, making the measurement of a μL-scale volume impossible. If an electronically controlled syringe pump had been utilized, then the actual pressure-volume relationship could have been recorded and compared with the estimated OR. However, this was not the primary goal of the study. Subject blood pressure was also not recorded.

Finally, it should be noted that reduced blood flow in the choroid does not necessarily translate to reduced blow flow in the retina or optic nerve head. Choroidal blood flow is influenced by innervation of the sympathetic and parasympathetic nervous system where retina and optic nerve head blood flow are regulated locally [2, 34, 36]. Both of these mechanisms are downstream of the split between the central retinal artery and the posterior ciliary arteries feeding the choroid suggesting that they may be isolated from each other [24, 36, 37]. For the data set used in this study, bupivacaine was used for a peribulbar block meaning autoregulation was likely impaired.

## Conclusion

Estimation of POBV provides a quick indication of ocular blood volume determined from IOP, OPA, and AL without the need to capture the ocular pressure waveform and compute its derivative. POBV is positively correlated with OPA, but negatively correlated with IOP. Higher IOP with lower OPA results in the lowest values of POBV. The OPA/IOP factor may also provide a useful clinical tool for evaluating changes in ocular blood flow in diseases with a vascular component, such as diabetic retinopathy and normal tension glaucoma. However, the Dynamic Contour Tonometer is no longer commercially available. The pneumatonometer (Model 30; Reichert Technologies, Inc) is a tonometer that is currently commercially available which measures and reports OPA, along with IOP. Future studies are warranted.

## Supporting information

S1 File DCT ICM human subject data English original format. (XLSX)

S2 File Description of variables DCT ICM. (XLSX)
